# Effects of Capsaicin on Growth Performance, Meat Quality, Digestive Enzyme Activities, Intestinal Morphology, and Organ Indexes of Broilers

**DOI:** 10.3389/fvets.2022.841231

**Published:** 2022-02-21

**Authors:** Zhihua Li, Jiaqi Zhang, Ting Wang, Jingfei Zhang, Lili Zhang, Tian Wang

**Affiliations:** College of Animal Science and Technology, Nanjing Agricultural University, Nanjing, China

**Keywords:** capsaicin, growth performance, meat quality, intestinal morphology, broiler

## Abstract

This experiment was conducted to investigate the effects of capsaicin (CAP) on growth performance, meat quality, digestive enzyme activities, intestinal morphology, and organ indexes of broilers. A total of 256 one-day-old Arbor Acre male broilers were randomly allocated into four treatments with eight replicates of eight birds, feeding a basal diet (control group), a basal diet supplemented with 2, 4, and 6 mg/kg CAP for 42 d, respectively. The growth performance, digestive enzyme activities of intestinal contents, small intestinal morphology, and organ indexes were measured at 21 and 42 d. The meat quality traits of breast muscles were determined at 42 d. The results showed dietary 4 mg/kg CAP supplementation decreased (*P* < 0.05) the feed to gain ratio (F/G) in the grower phase (22–42 d) and overall (1–42 d) compared with the control group, and 2 mg/kg CAP group also decreased (*P* < 0.05) the F/G from 1 to 42 d. Dietary 4 mg/kg CAP supplementation decreased (*P* < 0.05) the drip loss at 48 h and the pH_24h_ of breast muscles relative to the control group. Some digestive enzymes activities of jejunal and ileal contents were increased in the 2 and 4 mg/kg CAP groups compared with the control group both at 21 and 42 d. In addition, dietary 2 mg/kg CAP supplementation increased (*P* < 0.05) the relative weight of liver, jejunal villus height, villus width, and villous surface area at 21 d; The length of the jejunum segment and the relative weight of Bursa of Fabricius at 42 d in the 4 mg/kg CAP group were higher (*P* < 0.05) than the control group. In conclusion, dietary 2 or 4 mg/kg CAP supplementation decreased the F/G, improved meat quality, enhanced digestive enzyme activities, improved the jejunal development, and increased the relative liver and Bursa of Fabricius weight in broilers.

## Introduction

Poultry meat is in great demand in the global market for its high nutritional values, relatively low fat, and low price (1, 2). The meat quality genetic selection for larger breast muscles, along with increased gross body weight (BW) and rapid growth rate, may make modern chickens' immune function decline and susceptible to various stresses and diseases (3). Natural plant chemicals (active ingredients of most Chinese herbal medicines) are considered beneficial to organism health possibly through their anti-inflammatory, antioxidant, and prebiotic integration responses (4), thus resisting disease invasion and improving production performance. With the advent of the era of banning the use of antibiotics, research on plant extracts as a class of alternatives is in full swing.

Capsaicin (CAP), named as 8-methyl-N-vanilla base-6-nonene amide (C_18_H_27_NO_3_), is present at the highest level in capsaicinoids extracted from Chilli pepper (5). The transient receptor potential vanilloid 1 (TRPV1) ion channel is the receptor of CAP, whose discovery led to the 2021 Nobel Prize in Physiology or Medicine (6). For decades, CAP has shown the various biological functions through the TRPV1 pathway, including the anti-microbial, anti-obesity, anti-diabetes, anti-hypertension activities (7), anti-oxidation, anti-inflammation (8), and especially beneficial influences on the gastrointestinal system (9). The liver is the largest digestive gland in the body and the intestine is the main digestive tube. The two constitute the main digestive system, which is involved in the digestion of food and providing nutrients to the body for proper maintenance (10, 11). The previous studies demonstrated that dietary CAP supplementation had positive effects on the liver index, small intestinal absolute length, and villus structure (12–14) in rats and hamsters. Meanwhile, CAP could improve growth performance, antioxidant status, immune function, and meat quality in poultry (15, 16). What is more, CAP has a wide range of market sources with relatively low prices. The world's pepper production has increased from more than 12 million tons in 1993 to more than 31 million tons in 2013 (17). Therefore, CAP has clinical value and is a potential functional nutrient supplement for animals.

Previously, the studies mostly focused on the effects of capsicum oleoresin and a mixture of it with other plant extracts on live stocks, or mechanisms of biological functions of CAP (18, 19). However, researches on the application of purified CAP in poultry are still scarce. Besides, the beneficial effects of CAP on animals were related to its dosage and growth stage of animals (20), but the supplementation dosage of CAP was only one level in previous studies (15, 16). Thus, this study was carried out to investigate the effects of different levels of CAP supplementation on growth performance, meat quality, digestive enzyme activities, intestinal morphology, and organ indexes of broilers during the starter and grower stages, which would provide a further theoretical basis for its commercial application in the broiler feed.

## Materials and Methods

### Experimental Animals, Diets, and Design

A total of 256, male, 1-day-old Arbor Acres broilers were selected with 39.78 ± 0.16 g of average BW and randomly divided into four treatment groups including eight replicates with eight broilers per group. Broilers were, respectively, fed with a basal diet (control group) and a basal diet supplemented with either 100, 200, and 300 mg/kg CAP commercial product from the Guangzhou Leader Bio-Technology Co., Ltd., China, which accounts for 98% diluent (stearic acid) and 2% natural extracted CAP until 42 d of age. The dietary levels of CAP are correspondingly 2, 4, and 6 mg/kg diet. The CAP commercial product was mixed into the ingredients during the feed pelleting process. All broilers were provided a starter diet (pelleted and crumbled feed) from 1 to 21 d of age (starter stage) and a grower diet (pelleted feed) from 22 to 42 d of age (grower stage). The composition and nutrient levels of the basal diet for broilers were formulated based on the recommendation by the National Research Council (21), which are shown in Table 1. During the whole trial period, broilers were housed in wired cages (120 × 70 × 60 cm; 0.105 m^2^ per broiler) and had free access to water and feed. Broilers were kept in an environmentally controlled room at a temperature around 35°C for 5 d, which then gradually decreased to around 22°C and maintained it unchanged until the rest period, and supplied with a 23 h light and 1 h dark daily. Mortalities and healthy status of broilers were observed and recorded daily during the whole experimental time.

**Table 1:** Composition and nutrient level of the basal diet.

**Items**	**Days 1–21**	**Days 22–42**
**Ingredient (%)**
Corn	55.60	55.20
Soybean meal (CP, 44%)	29.00	24.00
Cottonseed meal	2.50	3.00
Wheat flour	4.00	4.00
Hydrolyzed feather meal	1.50	1.50
Dicalcium phosphate (16.5%)	0.90	0.80
Limestone powder	1.50	1.50
Bentonite	1.00	1.00
Soy oil	2.00	7.00
Premix^a^	2.00	2.00
Total	100.00	100.00
**Calculation of nutrients^b^**
Metabolizable energy, kcal/kg	2,894	3,212
Crude protein, %	21.50	19.51
Calcium, %	0.96	0.84
Total phosphorus, %	0.66	0.55
Lysine, %	1.45	1.40
Methionine, %	0.54	0.50
Threonine, %	0.91	0.80

a *Supplied per kilogram of diet: vitamin A, 11,500 IU; cholecalciferol, 3,500 IU; vitamin E, 30 mg; vitamin K3, 5 mg; thiamin, 3.38 mg; riboflavin, 9.0 mg; pyridoxine, 4.5 mg; vitamin B12, 0.025 mg; choline chloride, 800 mg; calcium pantothenate, 13 mg; niacin, 45 mg; biotin, 0.15 mg; folic acid, 1.20 mg; Mn (from manganese sulfate), 60 mg; Fe (from ferrous sulfate), 66.5 mg; Zn (from zinc sulfate), 88 mg; Cu (from copper sulfate), 8.8 mg; I (from calcium iodate), 0.70 mg; Se (from sodium selenite), 0.3 mg*.

b *The nutrient levels were as fed basis*.

### Growth Performance

The BW of the broilers was measured at 1, 21, and 42 d. The feed intake of the broilers in each replicate was recorded daily. The average daily gain (ADG), average daily feed intake (ADFI), and feed to BW gain ratio (F/G) were calculated.

### Sample Collection, Organ Indexes, and Carcass Traits

After feed deprivation for 12 h, one bird with a BW similar to the mean BW of its replicate was selected, weighed, and euthanized by exsanguination from each replicate of groups for the collection of breast muscle and intestine samples at 21 and 42 d. The left pectoralis major muscle sample was collected and stored at 4°C for the measurement of meat quality. The length of the jejunum (from the pancreatic loop to Meckel's diverticulum) and ileum (between Meckel's diverticulum and the caecal junction) was measured. Then the mid-jejunum and mid-ileum (1 cm) samples were collected and fixed in 4% paraformaldehyde for the morphological analysis. The jejunal and ileal contents samples (middle portion) were collected, snap-frozen in liquid nitrogen, and stored at −80°C for enzyme assays. Meanwhile, the liver, spleen, thymus, and Bursa of Fabricius were stripped and weighed to calculate the organ indexes. Furthermore, items on carcass traits were calculated after removing and weighing entrails and other relevant organs following the Terminology of Poultry Production Performance and Statistical Method of Measurement (NY/T823- 2004, China) at the end of the experiment.

### Breast Muscle Quality

The indexes of breast muscle quality were measured by the methods previously published (22, 23). Each index of each muscle sample was measured three times. Briefly, the breast muscle pH was detected at 45 min (pH_45min_) and 24 h (pH_24h_) using a pH meter (HI9125 portable waterproof pH/ORP meter, HANNA Instrument, Italy). The color of muscle was detected at 24 h postmortem by a colorimeter (Minolta CR-10, Konica Minolta Sensing, Japan) based on the CIELAB (international commission on illumination) system (L^*^ = lightness, a^*^ = redness, and b^*^ = yellowness). To measure the drip loss, the muscle samples were cut into 3 cm (length) × 2 cm (width) × 1 cm (thickness) sizes, which were weighed after hanging for 0 h (W1), 24 h (W2), and 48 h (W3) at 4°C. Drip loss at 24 h (%) = (W1–W2)/W1 × 100%, Drip loss at 48 h (%) = (W1– W3)/W1 × 100%. About 15 g of each regularly shaped muscle sample was prepared and weighed (W1), then placed at 4°C for 24 h, cooked in a water bath at 80°C until the internal temperature was up to 75°C, cooled, dried, and weighed again (W2). The amount of liquid separating during cooking was measured and calculated as a percentage of cooking loss by the following equation: Cooking loss (%) = (W1–W2)/W1 × 100%. The shearing force of each cooked meat was detected using a Digital Meat Tenderness Meter (C-LM3B, Northeast Agricultural University, Harbin, China) by shearing three different sections, the direction of which is vertical to the myofibers longitudinal axis.

### Digestive Enzyme Analysis

The jejunal and ileal contents were homogenized in the ice-cold physiological saline (wt/vol, 1:4) using an Ultra-Turrax homogenizer (Tekmar Co., Cincinnati, OH, USA) and then centrifuged at 3,500 × g for 10 min at 4°C. The supernatant was collected and stored at −80°C for further analysis. The protein concentration and digestive enzymes activities, including the amylase, lipase, and trypsin of the intestinal content homogenates were measured by spectrophotometric methods according to the manufacturers' instructions (Jiancheng Biological Engineering Research Institute, Nanjing, China).

### Morphological Measurements of the Small Intestine

The jejunal and ileal samples were fixed in 4% paraformaldehyde for 24 h and dehydrated in graded alcohol series (75, 85, 95, and 100%). Then the samples were immersed in xylene and embedded in paraffin. The tissue blocks were sectioned at 5 μm, dewaxed with xylene, rehydrated with alcohol, and finally stained with hematoxylin and eosin (hematoxylin for 1 min and 1% eosin for 10 s). Villus height and crypt depth of 10 well-oriented villi per segment were determined using an optical binocular microscope (Olympus BX5; Olympus Optical Co. Ltd, Tokyo, Japan) equipped with a digital camera (Nikon H550L; Nikon, Tokyo, Japan). Morphological parameters including villus length, crypt depth, and villus width were measured via the Dotslide software. The villous surface area was calculated following the previous work (24).

### Statistical Analysis

All statistical data were analyzed with the SPSS statistical software (Ver.22.0 for windows, SPSS Inc., Chicago, IL). The normality and homogeneity of variances of results were, respectively, evaluated by the Shapiro-Wilk test and Levene's test. The statistical significance of differences among the four groups was determined using a one-way analysis of variance accompanied by Tukey's *post hoc* test. Linear and quadratic effects of CAP supplementation were assessed using the polynomial contrasts. A pen (replicate) was an experimental unit for growth performance, and the individual broiler from each replicate was an experimental unit for other data. Results were shown as means with SEM. *P*-values <0.05 were considered to be significant.

## Results

### Growth Performance and Carcass Traits

As shown in Table 2, dietary CAP supplementation had no significant (*P* > 0.05) impacts on the ADG and ADFI during days 1 to 21, days 22 to 42, and during the whole period (days 1 to 42). Dietary supplementation with 4 mg/kg CAP decreased (*P* < 0.05) the F/G during days 22 to 42, compared with the control group. Dietary supplementation with 2 and 4 mg/kg CAP decreased (*P* < 0.05) the F/G during days 1 to 42, compared with the control group. Dietary CAP supplementation quadratically (*P* < 0.05) decreased the F/G from 22-42 d, and linearly (*P* < 0.05) and quadratically (*P* < 0.05) decreased the F/G from 1 to 42 d. There were no significant differences (*P* > 0.05) in the dressed, semi-eviscerated, eviscerated, breast muscle, and thigh muscle percentage among four treatments (Table 3).

**Table 2:** Effects of different levels of capsaicin on the growth performance of broilers.

**Item**	**Control**	**Dietary capsaicin level (mg/kg)**			**SEM**	***P*** **-value**		
		**2**	**4**	**6**		**ANOVA**	**Linear**	**Quadratic**
**Days 1–21**								
ADG (g/bird per day)	36.08	35.01	35.36	35.00	0.164	0.060	0.043	0.251
ADFI (g/bird per day)	45.80	44.04	45.33	44.02	0.313	0.091	0.133	0.701
F/G (g/g)	1.27	1.26	1.28	1.26	0.006	0.407	0.786	0.597
**Days 22–42**								
ADG (g/bird per day)	90.82	92.08	96.81	87.49	1.377	0.111	0.653	0.052
ADFI (g/bird per day)	151.71	144.41	147.80	140.51	1.815	0.156	0.063	0.999
F/G (g/g)	1.68^a^	1.57^ab^	1.53^b^	1.61^ab^	0.016	0.006	0.057	0.002
**Days 1–42**								
ADG (g/bird per day)	63.45	63.55	66.07	61.24	0.706	0.112	0.498	0.076
ADFI (g/bird per day)	99.94	95.51	98.07	93.34	1.152	0.194	0.095	0.949
F/G (g/g)	1.57^a^	1.50^b^	1.49^b^	1.52^ab^	0.010	0.007	0.038	0.004

*ADG, average daily gain; ADFI, average daily feed intake; F/G, feed to gain ratio. Values are presented as the mean with SEM (n = 8). Means with unlike superscript letters in the same row are significantly different (P < 0.05)*.

**Table 3:** Effects of different levels of capsaicin on carcass traits of broilers at 42 d.

**Item**	**Control**	**Dietary capsaicin level (mg/kg)**			**SEM**	***P*** **-value**		
		**2**	**4**	**6**		**ANOVA**	**Linear**	**Quadratic**
Dressed percentage (%)	90.35	90.97	90.42	90.70	0.227	0.772	0.806	0.716
Semi-eviscerated percentage (%)	82.55	81.91	82.97	83.20	0.311	0.499	0.293	0.501
Eviscerated percentage (%)	72.83	72.58	73.61	73.37	0.443	0.847	0.523	0.993
Breast muscle percentage (%)	30.07	33.10	31.34	30.44	0.476	0.104	0.875	0.038
Thigh muscle percentage (%)	11.10	10.56	10.22	10.32	0.158	0.201	0.061	0.303

*Values are presented as the mean with SEM (n = 8). Dressed percentage, semi-eviscerated percentage, and eviscerated percentage: Calculated as a percentage of live body weight. Breast muscle percentage and thigh muscle percentage: Calculated as a percentage of eviscerated carcass weight*.

### Meat Quality of Breast Muscle

As shown in Table 4, the pH_45min_ and pH_24h_ in the 6 mg/kg CAP group were lower (*P* < 0.05) than the 2 mg/kg CAP and control group, respectively. Dietary 4 mg/kg CAP supplementation significantly decreased (*P* < 0.05) pH_24h_ and drip loss at 48 h of breast muscle, compared with the control group. There were no significant differences (*P* > 0.05) in the L^*^, a^*^, and b^*^ values, cooking loss, and shearing force of breast muscles of broilers among the four groups. Dietary CAP supplementation linearly (*P* < 0.05) decreased the pH_45min_ and pH_24h_, and drip loss at 48 h of breast muscle.

**Table 4:** Effects of different levels of capsaicin on meat quality of breast muscle in broilers at 42 d.

**Item**	**Control**	**Dietary capsaicin level (mg/kg)**			**SEM**	***P*** **-value**		
		**2**	**4**	**6**		**ANOVA**	**Linear**	**Quadratic**
pH_45min_	6.52^ab^	6.73^a^	6.25^b^	6.32^b^	0.048	<0.001	0.002	0.365
pH_24h_	5.98^a^	5.91^ab^	5.88^bc^	5.82^c^	0.016	0.001	<0.001	0.966
L^*^	48.16	47.48	46.65	48.46	0.447	0.506	0.985	0.177
a^*^	3.51	3.34	3.40	3.37	0.134	0.973	0.768	0.803
b^*^	9.60	10.68	9.68	9.77	0.244	0.374	0.823	0.316
Drip loss at 24 h (%)	1.88	1.83	1.74	2.18	0.061	0.057	0.122	0.039
Drip loss at 48 h (%)	3.07^a^	3.12^a^	2.46^b^	2.70^ab^	0.088	0.016	0.016	0.561
Cooking loss (%)	26.00	24.35	27.63	29.78	0.771	0.071	0.031	0.194
Shearing force (N)	27.25	30.53	27.88	23.48	1.185	0.213	0.186	0.107

*Values are presented as the mean with SEM (n = 8). Means with unlike superscript letters in the same row are significantly different (P < 0.05). pH_45min_: pH value at 45 min; pH_24h:_ pH value at 24 h*.

L^*^, lightness;

a^*^, redness;

b^*^, yellowness.

### Digestive Enzyme Activities

The digestive enzyme activities for the jejunal and ileal contents of broilers were shown in Tables 5, 6. In the jejunal contents (Table 5), the lipase activity at 21 d in the broilers fed with the 2 and 4 mg/kg CAP diets was greater (*P* < 0.05) than those fed with the control diet. The trypsin activity of the 4 mg/kg CAP group was higher (*P* < 0.05) than the control group at 42 d. Dietary CAP supplementation linearly (*P* < 0.05) and quadratically (*P* < 0.05) increased the lipase activity at 21 d, as well as quadratically (*P* < 0.05) increased the trypsin activity at 21 and 42 d. In the ileal contents (Table 6), dietary 4 mg/kg CAP supplementation increased (*P* < 0.05) the lipase activity at 21 and 42 d compared with the control group. The amylase activity in the 2 mg/kg CAP group was higher (*P* < 0.05) than the control group at 42 d. Dietary CAP supplementation linearly (*P* < 0.05) and quadratically (*P* < 0.05) increased the lipase activity at 21 d, as well as quadratically (*P* < 0.05) increased the amylase and lipase activities at 42 d.

**Table 5:** Effects of different levels of capsaicin on the digestive enzyme activities of jejunal contents of broilers.

**Item**	**Control**	**Dietary capsaicin level (mg/kg)**			**SEM**	***P*** **-value**		
		**2**	**4**	**6**		**ANOVA**	**Linear**	**Quadratic**
**21 d**								
Amylase (U/mgprotein)	7.04	9.00	7.58	7.55	0.462	0.497	0.979	0.293
Lipase (U/gprotein)	381.15^b^	473.33^a^	472.73^a^	463.54^ab^	12.759	0.019	0.021	0.033
Trypsin (U/mgprotein)	4,096.86	4,804.43	4,888.41	4,612.78	119.492	0.077	0.113	0.036
**42 d**								
Amylase (U/mgprotein)	7.54	9.28	8.60	8.17	0.484	0.658	0.787	0.286
Lipase (U/gprotein)	435.16	502.33	518.63	482.15	14.941	0.226	0.237	0.086
Trypsin (U/mgprotein)	4,104.88^b^	4,836.4^ab^	5,023.48^a^	4,538.53^ab^	126.873	0.049	0.164	0.014

*Values are presented as the mean with SEM (n = 8). Means with unlike superscript letters in the same row are significantly different (P < 0.05)*.

**Table 6:** Effects of different levels of capsaicin on the digestive enzyme activities of ileal contents of broilers.

**Item**	**Control**	**Dietary capsaicin level (mg/kg)**			**SEM**	***P*** **-value**		
		**2**	**4**	**6**		**ANOVA**	**Linear**	**Quadratic**
**21 d**								
Amylase (U/mgprotein)	8.51	9.30	8.96	7.92	0.535	0.832	0.672	0.416
Lipase (U/gprotein)	394.36^b^	494.68^ab^	510.10^a^	477.69^ab^	15.000	0.022	0.034	0.019
Trypsin (U/mgprotein)	4,577.07	4,718.93	4,797.96	4,541.24	116.288	0.862	0.979	0.416
**42 d**								
Amylase (U/mgprotein)	7.19^b^	11.10^a^	10.02^ab^	8.94^ab^	0.504	0.033	0.315	0.011
Lipase (U/gprotein)	417.69^b^	505.73^ab^	520.17^a^	482.68^ab^	13.233	0.023	0.057	0.013
Trypsin (U/mgprotein)	4,488.42	5,105.69	4,929.34	4,620.11	130.237	0.329	0.851	0.083

*Values are presented as the mean with SEM (n = 8). Means with unlike superscript letters in the same row are significantly different (P < 0.05)*.

### The Liver Index and Length of the Small Intestine

In the present study, dietary 2 mg/kg CAP supplementation increased (*P* < 0.05) the relative liver weight of broilers at 21 d (Table 7), compared with the control group. There was no significant difference (*P* > 0.05) in the relative liver weight of broilers among the four groups at 42 d. As presented in Table 7, dietary CAP supplementation had no significant effects (*P* > 0.05) on the length of jejunum and ileum in broilers at 21 d. Dietary 4 mg/kg CAP supplementation increased (*P* < 0.05) the length of jejunum and ileum at 42 d, compared with the control group and broilers fed with 6 mg/kg CAP, respectively. Dietary CAP supplementation quadratically (*P* < 0.05) increased the length of jejunum and ileum at 42 d.

**Table 7:** Effects of different levels of capsaicin on the liver index and the length of the small intestine of broilers.

**Item**	**Control**	**Dietary capsaicin level (mg/kg)**			**SEM**	***P*** **-value**		
		**2**	**4**	**5**		**ANOVA**	**Linear**	**Quadratic**
**21 d**								
Liver weight/BW(g/kg)	25.39^b^	29.37^a^	28.21^ab^	28.49^ab^	0.524	0.034	0.064	0.060
Length of jejunum (cm)	51.75	53.23	54.76	53.41	0.810	0.650	0.386	0.401
Length of ileum (cm)	40.28	40.10	43.60	42.61	0.905	0.449	0.207	0.825
**42 d**								
Liver weight/BW(g/kg)	24.56	23.56	25.01	22.94	0.584	0.607	0.527	0.654
Length of jejunum (cm)	65.13^b^	74.63^ab^	79.00^a^	68.88^ab^	1.659	0.010	0.231	0.002
Length of ileum (cm)	47.00^ab^	51.50^ab^	53.00^a^	45.63^b^	0.965	0.011	0.728	0.001

*Values are presented as the mean with SEM (n = 8). Means with unlike superscript letters in the same row are significantly different (P < 0.05)*.

### Small Intestinal Morphology

**Figure 1:**
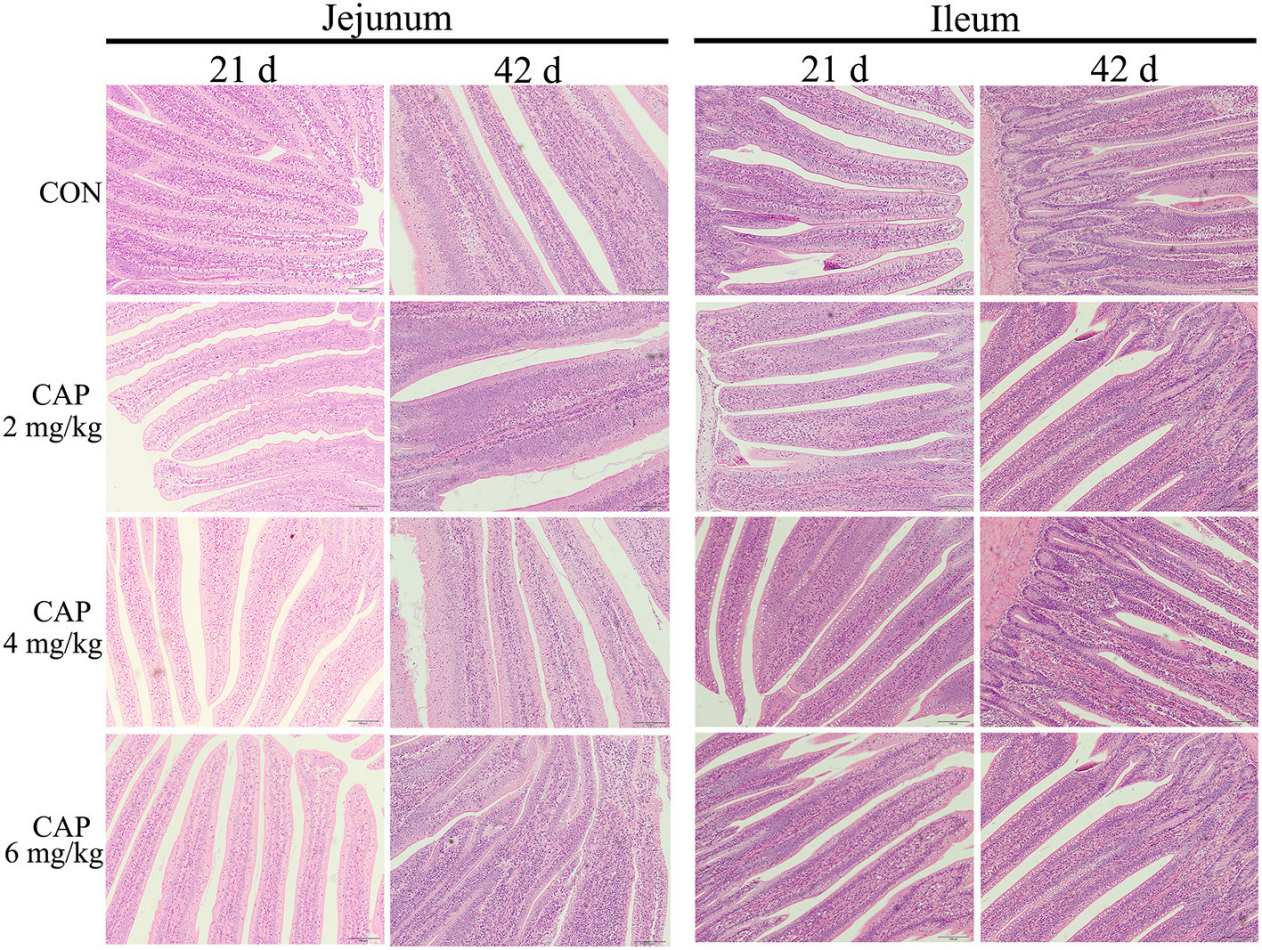
The jejunum and ileum histological morphology of broilers (hematoxylin and eosin). CON, broilers were fed with a basal diet; CAP 2, CAP 4, and CAP 6 mg/kg, broilers were fed with a basal diet supplemented with 2, 4, and 6 mg/kg capsaicin, respectively. Scale bars = 100 μm.

The morphologic analysis of the small intestine in broilers was shown in Tables 8, 9, and Figure 1. Dietary supplementation with 2 mg/kg CAP could increase (*P* < 0.05) the villus height, width, and surface area of jejunum at 21 d, compared with the control group (Table 8). Dietary CAP supplementation quadratically (*P* < 0.05) increased the jejunal villus height, villus width, and surface area at 21 d. There were no significant differences (*P* > 0.05) in the villus height, crypt depth, villus width, villus height/crypt depth, and villous surface area of jejunum at 42 d, and ileum at 21 and 42 d (Tables 8, 9). As shown in Figure 1, the structure of villi was basically integral in the representative samples of jejunum and ileum in four groups.

**Table 8:** Effects of different levels of capsaicin on the jejunal morphology of broilers.

**Item**	**Control**	**Dietary capsaicin level (mg/kg)**			**SEM**	***P*** **-value**		
		**2**	**4**	**6**		**ANOVA**	**Linear**	**Quadratic**
**21 d**								
Villus height (μm)	988.69^b^	1,216.87^a^	1,084.06^ab^	973.09^b^	31.696	0.013	0.452	0.004
Crypt depth (μm)	233.18	272.93	241.05	250.58	6.594	0.162	0.720	0.240
Villus width (μm)	122.61^b^	179.94^a^	139.43^b^	142.29^b^	5.970	0.001	0.640	0.005
Villus height/crypt depth (μm/μm)	4.35	4.46	4.56	3.92	0.149	0.473	0.393	0.227
Villous surface area ( × 10^3^ μm^2^)	191.39^b^	329.52^a^	244.84^ab^	231.71^ab^	16.115	0.010	0.760	0.009
**42 d**								
Villus height (μm)	1,622.78	1,685.03	1,750.36	1,558.55	41.445.	0.420	0.735	0.141
Crypt depth (μm)	332.37	328.63	342.16	329.19	9.026	0.885	0.963	0.812
Villus width (μm)	244.02	244.67	211.97	223.23	7.195	0.301	0.148	0.711
Villus height/crypt depth (μm/μm)	4.93	5.29	5.14	4.74	0.147	0.602	0.604	0.222
Villous surface area (× 10^3^ μm^2^)	622.37	654.65	587.25	555.86	27.993	0.654	0.313	0.587

*Values are presented as the mean with SEM (n = 8). Means with unlike superscript letters in the same row are significantly different (P < 0.05)*.

**Table 9:** Effects of different levels of capsaicin on the ileal morphology of broilers.

**Item**	**Control**	**Dietary capsaicin level (mg/kg)**			**SEM**	***P*** **-value**		
		**2**	**4**	**6**		**ANOVA**	**Linear**	**Quadratic**
**21 d**								
Villus height (μm)	817.14	817.06	810.61	756.84	18.831	0.643	0.293	0.497
Crypt depth (μm)	229.00	217.24	196.36	194.97	6.146	0.136	0.027	0.657
Villus width (μm)	141.09	145.44	141.76	122.33	4.707	0.315	0.163	0.214
Villus height/crypt depth (μm/μm)	3.58	3.78	4.13	3.95	0.090	0.148	0.064	0.268
Villous surface area (× 10^3^ μm^2^)	182.77	185.05	180.18	146.13	6.778	0.132	0.056	0.167
**42 d**								
Villus height (μm)	1,250.53	1,141.65	1,242.32	1,060.76	34.650	0.164	0.126	0.586
Crypt depth (μm)	223.17	236.12	250.96	221.05	6.705	0.385	0.889	0.124
Villus width (μm)	170.97	191.91	169.93	170.43	4.349	0.208	0.534	0.235
Villus height/crypt depth (μm/μm)	5.62	4.95	5.00	4.81	0.150	0.237	0.081	0.418
Villous surface area (× 10^3^ μm^2^)	334.80	342.68	338.95	285.50	13.232	0.399	0.214	0.259

*Values are presented as the mean with SEM (n = 8). Means with unlike superscript letters in the same row are significantly different (P < 0.05)*.

### The Immune Organ Indexes

There were no significant differences (*P* > 0.05) in the relative spleen and thymus weight of broilers at 21 and 42 d among the four groups (Table 10). The relative Bursa of Fabricius weight of broilers at 21 and 42 d fed with 2 mg/kg CAP was higher (*P* < 0.05) than the 6 mg/kg CAP group and other three groups, correspondingly. Dietary CAP supplementation quadratically (*P* < 0.05) increased the relative Bursa of Fabricius weight at 21 and 42 d.

**Table 10:** Effects of different levels of capsaicin on the immune organ indexes of broilers (g/kg).

**Item**	**Control**	**Dietary capsaicin level (mg/kg)**			**SEM**	***P*** **-value**		
		**2**	**4**	**6**		**ANOVA**	**Linear**	**Quadratic**
**21 d**								
Spleen weight/BW	0.84	0.87	0.91	0.86	0.035	0.915	0.754	0.583
Thymus weight/BW	4.33	4.40	4.74	3.82	0.151	0.197	0.368	0.102
Bursa of Fabricius weight/BW	1.92^ab^	2.40^a^	2.05^ab^	1.82^b^	0.080	0.049	0.325	0.024
**42 d**								
Spleen weight/BW	0.96	0.96	0.97	0.93	0.031	0.959	0.759	0.743
Thymus weight/BW	2.31	2.51	2.48	2.34	0.061	0.610	0.897	0.190
Bursa of Fabricius weight/BW	0.84^b^	1.19^a^	0.93^b^	0.91^b^	0.039	0.026	0.856	0.009

*BW, body weight. Values are presented as the mean with SEM (n = 8). Means with unlike superscript letters in the same row are significantly different (P < 0.05)*.

## Discussion

CAP, a major pungent hydrophobic alkaloid of capsicum fruits, shows several biological activities, including antimicrobial activity, improving digestive function, antioxidation, anti-inflammation, and so on, which is conducive to livestock production (7, 25). The presented results showed that dietary 2 and 4 mg/kg CAP had more distinct effects than the dietary 6 mg/kg CAP supplementation, possibly due to less irritation. Meanwhile, 2 and 4 mg/kg CAP groups are more cost-effective than the 6 mg/kg CAP group.

In the present study, dietary 2 and 4 mg/kg CAP supplementation could decrease the F/G, which might improve the feed efficiency, thereby reducing the breeding costs. This may be partly because dietary CAP supplementation could increase the digestive enzymes (15, 26, 27), bile acids (14), and endogenous cholecystokinin (28) in the gut, which are required for the efficient digestion of nutrients. In accordance with it, we found that dietary 2 or 4 mg/kg CAP supplementation could enhance the lipase activity of jejunal and ileal contents, trypsin activity of jejunal contents, and amylase activity of ileum contents. These endogenous digestive enzymes play important roles in the whole digestion process. The amylase, lipase, and trypsin are good for the decomposition, digestion, and absorption of carbohydrates, protein, and lipid, respectively (29). Similarly, feeding the mixture of 5% carvacrol, 3% cinnamaldehyde, and 2% capsicum oleoresin at 100 mg/kg improved the feed efficiency by 9.8% in Ross 308 male broiler chickens (30). Interestingly, it was reported no differences in feed efficiency were observed in the leghorn chicks fed with diets containing 5 and 20 mg/kg CAP (31). This controversy may be due to the level and composition of capsaicin preparation in the basal diet, diet composition, broiler types, and feeding management. The CAP in the present study was processed by microencapsulation technology which could help to decrease the irritating effect and control its release throughout the gastrointestinal tract (25).

The physical characteristics of meat quality including pH, meat color, water holding capacity, tenderness, and so on determine the acceptability, storage, and processability of meat products. The accumulation of lactic acid in the muscles caused by glycogen degradation leads to a pH decline at 24 h (32). The pH value can be associated with color, cooking loss, tenderness, shelf-life, and other traits (33). In the present study, dietary 4 mg/kg CAP supplementation reduced the pH_24h_ value of muscle within a normal range (5.7 < pH_24h_, not low enough to promote the formation of the pale, soft, and exudative meat), which may be beneficial to prolong the shelf life of muscle (32, 34), as it may retard meat spoilage through inhibiting microbes growth (33). In addition, meat color is the appearance and manifestation of the physiological, biochemical, and microbiological changes of muscle, which can influence consumer acceptance of poultry meat (35). Drip loss and cooking loss reflect the water-holding capacity (WHC). Dietary 4 mg/kg CAP supplementation also decreased the drip loss at 48 h, which may increase the water content of muscles and enhance tenderness and juiciness, thus improving the meat quality (2). The shearing force partly reflects the tenderness of the meat. There were no significant differences in the meat color, cooking loss, and shearing force of breast meat after CAP treatment. Theoretically, the higher light value of meat is accompanied with lower pH, higher moisture, and lower WHC (35). However, the pH value has little relationship to other parameters, such as meat color and drip losses in our study. These irrelevances maybe because the meat quality of breast muscle measured in this trial is within the normal range, or there may be some other factors affecting these meat quality traits. The above indicates that dietary MC supplementation could improve the meat quality by decreasing the pH and drop losses. What's more, the main target of commercial broiler chicken production is achieving high-yield and high-quality muscles, especially breast muscle, which is considered the most valuable piece of the chicken carcass and greatly affected by the breed (36). In the present study, dietary MC supplementation had no significant influences on the carcass trait.

To further understand the effects of CAP on modulating nutrient digestion and absorption, we measured the liver and small intestinal development. In the present study, dietary 2 mg/kg CAP supplementation increased the relative liver weight at 21 d, which is beneficial to enhance digestive function. It was also reported that a plant extract mixture including 1.98 mg/kg CAP increased the liver weight of broilers at 42 d (27). In addition, histologically, longer and wider villi, and larger villous surfaces of the small intestine indicate an increase in feed efficiency and growth-promoting efficiency (37). In general, villi are the longest in the jejunum where most nutrients are digested and absorbed. Previous studies have shown that dietary pungent spices supplementation increased the length of the small intestine in rats (38) and the length and perimeter of microvilli, which contributes to an increase in the absorption surface of the small intestine, thereby improving the bioavailability of micronutrients (12). Similarly, in our study, dietary 2 and 4 mg/kg CAP supplementation also increased the length of jejunum and ileum at 42 d and enhanced the villus height, villus width, and villous surface area in the jejunum at 21 d, suggesting CAP could promote small intestine development and improve the utilization of nutrients in broilers. These positive effects of CAP on the liver and small intestine may be one of the reasons for its reduced F/G.

The immune organ index is commonly measured to assess immunity in poultry (34). The Bursa of Fabricius is vital to the normal development of B lymphocytes and antibody production (39). Furthermore, the Bursa of Fabricius may expand the antibody-producing apparatus by promoting the contact between intestinal antigens and lymphoid tissue of the organ wall (40). Our studies showed dietary 2 mg/kg CAP supplementation increased the relative Bursa of Fabricius weight, which is directly in line with the previous finding (15), suggesting CAP is conducive to the improvement of immune function.

## Conclusions

Dietary 2 or 4 mg/kg CAP supplementation could reduce the F/G, improve meat quality, increase digestive enzymes of small intestinal contents and improve the liver, small intestine, and immune organ development of broilers. Therefore, CAP can be used as a functional additive for broilers.

## Data Availability Statement

The original contributions presented in the study are included in the article/supplementary material, further inquiries can be directed to the corresponding author/s.

## Ethics Statement

The animal study was reviewed and approved by Animal Care and Use Committee of Nanjing Agricultural University.

## Author Contributions

ZL and TiaW conceived and designed the experiment. ZL, JiaZ, and TinW performed the experiment. ZL and JinZ processed the data. ZL prepared and drafted the manuscript. LZ and TiaW revised the manuscript. All authors reviewed the final manuscript.

## Funding

This work was supported by the National Natural Science Foundation of China (nos. 31772634 and 32172775).

## Conflict of Interest

The authors declare that the research was conducted in the absence of any commercial or financial relationships that could be construed as a potential conflict of interest.

## Publisher's Note

All claims expressed in this article are solely those of the authors and do not necessarily represent those of their affiliated organizations, or those of the publisher, the editors and the reviewers. Any product that may be evaluated in this article, or claim that may be made by its manufacturer, is not guaranteed or endorsed by the publisher.
